# Dietary Protein Intake, Meat Consumption, and Dairy Consumption in the Year Preceding Pregnancy and During Pregnancy and Their Associations With the Risk of Gestational Diabetes Mellitus: A Prospective Cohort Study in Southwest China

**DOI:** 10.3389/fendo.2018.00596

**Published:** 2018-10-11

**Authors:** Yi Liang, Yunhui Gong, Xiao Zhang, Dagang Yang, Danqing Zhao, Liming Quan, Rong Zhou, Wei Bao, Guo Cheng

**Affiliations:** ^1^West China School of Public Health and Healthy Food Evaluation Research Center, Sichuan University, Chengdu, China; ^2^Department of Obstetrics and Gynecology, West China Second University Hospital, Key Laboratory of Birth Defects and Related Diseases of Women and Children of Ministry of Education, Sichuan University, Chengdu, China; ^3^Department of Obstetrics and Gynecology, Longquanyi District of Chengdu Maternity and Child Health Care Hospital, Chengdu, China; ^4^Department of Clinical Nutrition, Affiliated Hospital of Guizhou Medical University, Guiyang, China; ^5^Department of Obstetrics and Gynecology, Affiliated Hospital of Guizhou Medical University, Guiyang, China; ^6^Department of Epidemiology, College of Public Health, University of Iowa, Iowa City, IA, United States; ^7^West China School of Public Health and State Key Laboratory of Biotherapy and Cancer Center, Sichuan University, Chengdu, China

**Keywords:** gestational diabetes mellitus, insulin resistance, protein intake, longitudinal cohort, meat consumption, dairy consumption

## Abstract

**Background:** Gestational diabetes mellitus (GDM) has become a public health problem in China.

**Objective:** To examine the association of dietary protein intake before and during pregnancy with the risk of GDM.

**Design:** Dietary intake before pregnancy and during the first and second trimesters of pregnancy was assessed using food frequency questionnaires in a prospective cohort of pregnant women. To screen GDM, participants underwent an OGTT test during 24–28 weeks of gestation. Cox proportional hazards were used to estimate RRs and 95% CIs for the associations between tertiles of dietary protein and the source of protein intake in different time windows with GDM status.

**Results:** Higher intake of total protein [RR (95% CI): 1.92 (1.10–3.14), *p* for trend = 0.04] or animal protein [1.67 (1.19–2.93), *p* for trend = 0.03] in mid-pregnancy was associated with higher risk of GDM. Vegetable protein intake before or during pregnancy was not related to GDM risk (*p* for trend > 0.05). Moreover, in the mid-pregnancy, participants with higher meat consumption or dairy consumption had a higher risk of GDM.

**Conclusion:** Our study indicated that higher dietary intakes of total protein and animal protein in mid-pregnancy were associated with an increased risk of GDM among pregnant Chinese women.

## Introduction

Gestational diabetes mellitus (GDM) is a common complication of pregnancy characterized by glucose intolerance with onset or first recognition during pregnancy (1). For mothers, GDM is not only associated with adverse perinatal outcomes (2), it is also related to a higher risk of GDM during subsequent pregnancies (3, 4) and type 2 diabetes and premature cardiovascular disease in the medium and long term (5). For offspring, GDM pregnancy confers a greater risk of developing obesity, diabetes, hypertension, cardiovascular disease in youth, and adult life (6, 7). According to the diagnostic criteria of 2011 issued by the Ministry of Health (MOH) China (8), which endorsed the new criteria by the IADPSG (1), the prevalence of GDM in China is 17.5% in 2013 (9), which is higher than the prevalence in Europe and the USA (10, 11). Given the substantial relevance of GDM for medium and long-term health of both mother and offspring, it is crucial to identify modifiable risk factors which contribute to GDM prevention among Chinese population.

GDM is a disease of disturbed glucose homeostasis (1). In pregnancy, the sensitivity of insulin is reduced by 50–70% (12), while compensatory increase in insulin secretion is 2–2.5 times to maintain normal blood glucose level (13). Consequently, pregnancy represents a physiological state of insulin resistance. Evidence supports that chronic insulin resistance is a central component of the pathophysiology of GDM (14). Dietary proteins might play a vital role in the pathogenesis of insulin resistance, acting as gluconeogenic precursors thereby stimulating hexosamine biosynthesis, or activating the mTOR-signaling pathway (15, 16). Hence, an understanding of the role of the dietary protein intake on the GDM development may have important public health implications. Several studies have found that a diet during mid-pregnancy with a higher protein intake among Asian women (17, 18) or higher protein intake among US women before pregnancy (19) is related to higher GDM risk, while a previous study among Canada women did not find the relationship between protein intake and GDM (20). The reasons for these inconsistent results may be due to the various sample sizes [205 (20) to 15,294 (19)], the focused observation time window, the diagnostic criteria for GDM [clinical examination: WHO standard (1999) (17), IADPSG standard (2010) (18), NDDG standard (1979) (20), and self-reported (19)] or the ethnicity of the research participants [Asians (17, 18), Americans (19), Canadians (20)].

To date, all cohort studies analyzing the link between maternal individual nutrients and GDM risk addressed dietary intake only in one time window, i.e., either before pregnancy (19, 21–28) or during pregnancy (29–31). However, individual dietary protein intake level may change during pregnancy (32). An increase in the intake of protein rich foods during pregnancy was reported in UK (33), Portugal (34), Switzerland (35), Hungary (36), and Asia (37), especially a remarkable increase in protein intake during mid-pregnancy was observed among Portuguese (34) and Swedes (35). In terms of public health, it would thus be intriguing to clarify whether there is a critical time window for dietary protein intake, taking into consideration pre-pregnancy intake and intake across the two pregnancy trimesters preceding the diagnosis of GDM.

Therefore, using data from the Nutrition in Pregnancy and Growth in Southwest China (NPGSC) prospective cohort study, we examined whether dietary protein intake during potentially critical periods, i.e., the year preceding pregnancy, 1st or early 2nd trimester were associated with GDM risk.

## Materials and methods

### Study sample

We used data from the NPGSC study, which is a prospective cohort study initiated in January 2014 to investigate the relevance of maternal nutrition before and during pregnancy on health outcomes of mother (e.g., gestational diabetes mellitus) and child (e.g., birth weight, birth length, and body composition development in childhood). Using a sampling design stratified by urban and rural locations, a representative sample of pregnant women was drawn from public hospitals with obstetric services in Southwest China (Sichuan Province, Yunnan Province, and Guizhou Province). Within each urban area 2 public hospitals were randomly selected, while 2–3 public hospitals were randomly selected within each rural area. In total, 27 study centers (12 urban hospitals and 15 rural hospitals) were included until December 2017. The study was approved by the Ethics Committee of Sichuan University.

At each center, pregnant women were invited during their first visit for routine ultrasound examination at gestational weeks 9–11. To facilitate follow-up, only women who had lived in their current residence for at least 2 years were eligible to participate. The overall response rate was 91.2%.

**Figure 1:**
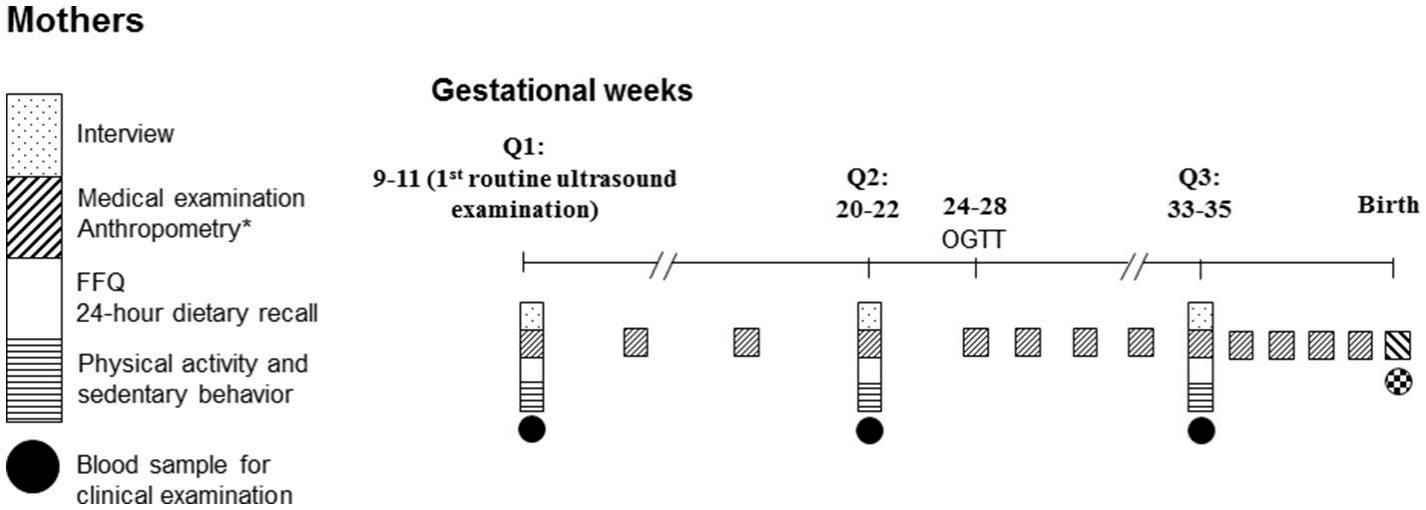
The study examination schedule.

Data collection of NPGSC study was performed 3 times before birth (the first routine ultrasound examination, Q1; gestational weeks 20–22, Q2; gestational weeks 33–35, Q3) and 8 times in infancy and childhood (Figure 1). At Q1, approached by trained interviewers and local nurses, each woman was asked to complete a self-administered questionnaire to collect information on her birth characteristics, demographic characteristics, medical history, lifestyle (e.g., smoking behavior, alcohol consumption, tea/coffee consumption), employment, annual family income, and family history of chronic diseases. In addition, participants were interviewed by trained investigators in a face-to-face interview with respect to their diet [one food frequency questionnaire (FFQ) covering the consumption over the past 12 months before pregnancy and one 24-h dietary recall addressing intake over the past 24 h] and physical activity (one questionnaire covering physical activity and sedentary behavior over the past 12 months before pregnancy and from the start of the pregnancy, separately). At Q2 and Q3, one 24-h dietary recall each inquiring intake over the past 24 h and one FFQ addressing consumption during the previous 12 weeks, as well as one physical activity questionnaire were administeredby trained investigators in face-to-face interviews.

Maternal anthropometrics measures (body weight before pregnancy and during pregnancy), clinical measures, as well as information on current and past pregnancy outcomes, complications, and infant abnormalities recorded in the Medical Birth Registry were linked to the study database. Furthermore, anthropometric measures of offspring and information of cognitive development test which have been followed in infancy and childhood and recorded in health registries were linked to the study database. At Q1, all participants provided written informed consent for all examinations as well as for linkage of their data from the Medical Birth Registry and the data of their offspring from the health registries.

**Figure 2:**
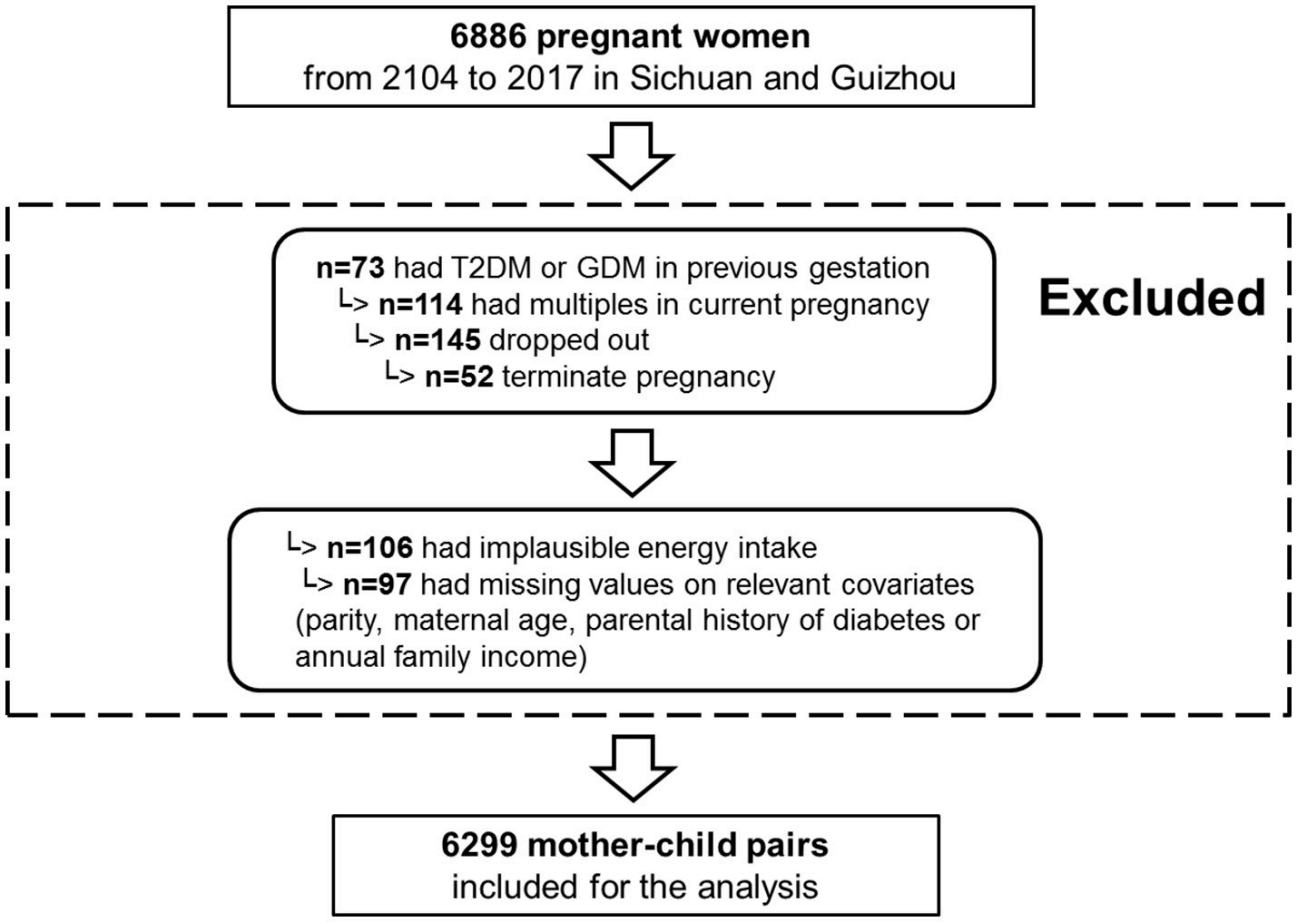
Flowchart for the study sample.

From 2014 to 2017, 1,0126 pregnant women were recruited in the NPGSC Study. For the current analysis, we used information on diet, anthropometry and clinical measures collected between 2014 and 2017 from mothers living in the Sichuan Provence and Guizhou Province (18 study centers: 8 urban hospitals and 10 rural hospitals). The Ministry of Health China recommends screening by fasting plasma glucose (FPG) test at the first prenatal visit to rule out previously undiagnosed preexisting diabetes. Of the 6,886, 73 women with preexisting diabetes mellitus before pregnancy were excluded. To be included in this analysis, participants had to have delivered a live, singleton baby and to have answered the first general questionnaire resulting in 6,686 women. Of them, 6,502 had provided three FFQs for the dietary intakes before pregnancy, at 1st trimester, or at 2nd trimester. We further excluded women with an implausible energy intake (< 500 or ≥3,500 kcal/day) (38) (*n* = 106), as well as those with missing information on parity (*n* = 36), maternal occupation (*n* = 23), or annual family income (*n* = 38). In total, 6,299 women with complete information were included in this analysis (Figure 2).

### Nutrition assessment

Since only FFQ covering consumption over the past 12 months before pregnancy can be used for the estimation of dietary intake before pregnancy, the present analyses is based dietary data collected by FFQs. At each Q1, Q2, and Q3, a modified validated 128-item FFQ (39) was used to inquire how often, on average (never to ≥5 times/d) the participants had consumed the respective food groups [e.g., white rice, brown rice, red rice, wheat noodle, steamed bread, bread, whole grain foods, potatoes, cakes, vegetables, fruits, subtropical fruits, dairy and dairy products, soybeans, and its products, nuts (walnuts, almonds, cashews, peanuts, and other nuts), meat (pork, beef, chicken, or lamb), eggs, fish and shrimp, and beverages (including drinking water, mineral water, tea and herbal tea, lemonades, fruit drinks (diluted and sugar-sweetened fruit juices), ice teas, soft drinks, and sports drinks)], using standard serving sizes. The participants were offered a range of different serving sizes for each food and beverage item. To each foods item, vegetable protein, and animal protein were categorized. In our study, the most important determinant of animal protein were meats, fish and shrimp, eggs, dairy and dairy products, and for vegetable protein were beans (sum of soybeans and its products), and nuts. Visual aids such as standard serving bowls, plates and glasses were displayed to the participants to improve the accuracy of the estimated portion sizes. The frequency and amount of consumption of each food or beverage per unit of time were converted into food consumption per day. Total energy and nutrient intakes were calculated using the continuously updated in-house nutrient database (40) reflecting the composition of Chinese Foods (41). This nutrient database includes any food item ever recorded in previous studies conducted and is based on information from standard nutrient tables, product labels (e.g., most convenience foods) or recipe simulation based on the labeled ingredients and nutrients (e.g., commercial mixed dishes).

### Diagnostic criteria for GDM

At 24–28 weeks of gestation, participants underwent a 2-h 75-g oral-glucose-tolerance test (OGTT) after an overnight fast, and venous plasma glucose levels at 0, 1, and 2 h were measured. A diagnosis of GDM was made if any one of the following values was met or exceeded: 0 h (fasting), ≥5.1 mmol/L; 1 h, ≥10.0 mmol/L; and 2 h, ≥8.5 mmol/L, according to the diagnostic criteria for GDM recommended by the Chinese Ministry of Health (8). The diagnostic criteria were in line with the criteria proposed by the International Association of Diabetes and Pregnancy Study Groups.

#### Quality control

All study laboratories successfully completed a standardization and certification program. The coefficient of variation within and between study laboratories was < 5% for each marker. All laboratory equipment was calibrated and blinded duplicate samples were used. All data were double entered into the database. Participants were informed about all clinical data within 36 h after collection.

### Other clinical examinations

Self-reported pregravid weight was recorded on the day of registration. Body weight was measured with an ultrasonic meter (Dingheng, Zhengzhou, China) to the nearest 100 g by trained nurses according to standard procedures at enrollment and at regular intervals (in 4-week intervals from enrolment to week 25, every 2-week until week 33 weekly thereafter) to birth (Figure 1). These information and maternal height (measured to the nearest 0.1 cm by trained nurses at enrollment) were recorded in the Medical Birth Registry.

For all participants, gestational age (GA) was assessed during the first ultrasound scan (Eub 5500, Hitachi; Eub 7500, Hitachi; Logiq E9, GE) on the day of registration, which was conducted in a standard manner by trained ultrasonographers. GA was estimated by combining ultrasonography data with self-report on the last menstrual period: if both measures were available and there was agreement (±14 days) self-report data was used, otherwise, ultrasound data were used.

### Statistical analysis

SAS® procedures (version 9.2, SAS Inc., Cary, NC) were used for all data analyses. All analyses were performed with a significance level at *p* < 0.05.

Because we were interested in the critical time window regarding the relevance of dietary protein intakes for GDM onset, we conducted separate analyses using dietary data collected before pregnancy and during the 1st trimester and 2nd trimester of pregnancy. Dietary protein intakes in these 3 different periods were expressed each as residuals from their regression on energy intake.

Energy-adjusted residuals of dietary protein intakes were grouped into tertiles to illustrate their associations with the risks of GDM using Cox proportional hazards analysis. In the basic models, dietary protein intakes were the independent predictors. The following variables potentially affecting these associations were considered: gestational age, age, parity, location (urban/rural), family history of diabetes, maternal education level (12 or more years of schooling; yes/no), maternal occupation (no, yes: part-time worker, or full-time worker), monthly personal income (< 3,000 CNY, 3,000–6,000 CNY, >6,000 CNY), and pregravid BMI, physical activity, smoking/passive smoking before or during pregnancy (never, past, current: 1–15, 16–24, or >24 cigarettes/d), alcohol consumption before or during pregnancy (0, 0.1–9.9, 10.0–19.9, 20.0–29.9, ≥30 g/d), gestational weight gain during pregnancy, and intakes of carbohydrate and fat at the same time point of dietary protein. Each potential confounder was initially considered separately and included if it substantially modified the association of dietary protein with GDM or significantly predicted the outcome variable. Thus, maternal age and parity were retained in model 2. In a further step, we adjusted for parental history of diabetes (yes or no), current smoking (combination of passive smoking and active smoking, yes or no), polycystic ovarian syndrome (PCOS, yes or no), family income, total energy intake, fat intake, carbohydrate intake and physical activity. Animal protein and vegetable protein were mutually adjusted for one another. Relative risk (RR) were calculated for the respective tertiles and a test for trend was performed using the respective continuous variables.

## Results

### General characteristics

Maternal characteristics are presented in Table 1. The mean age of participants was 26.5 years and the mean pre-gravid BMI was 20.7 kg/m^2^. GDM was diagnosed in 1,203 of 6,299 pregnancies. 23.7% of our participants reported family history of diabetes, 41.8% of them had less than a college education.

**Table 1:** General characteristics^a^ of the study participants.

**Characteristics**	**Values (*n* = 6,299)**
Urban [*n* (%)]	3,030 (48.1)
Maternal age (years)	26.5 (3.8)
Pregravid BMI (kg/m^2^)	20.7 (2.4)
Gestational diabetes mellitus [*n* (%)]	1,203 (19.1)
Single mother [*n* (%)]	157 (2.5)
High education level^b^ [*n* (%)]	3,666 (58.2)
Moderate personal monthly income^c^ [*n* (%)]	3,281 (52.1)
**PREVIOUS MEDICAL HISTORY AND FAMILY HISTORY**	
Polycystic ovarian syndrome [*n* (%)]	119 (1.9)
Primiparous (*n*, %)	4,585 (72.8)
Family history of diabetes [*n* (%)]	1,492 (23.7)

a *Values are means (SD) or frequencies*.

b *School education at least 12 years*.

c *Personal income per month at least ≥3,000 CNY (Chinese Yuan), which is an average level among the general population in Southwest China*.

Women in the 2nd trimester had gained more weight compared with women in the 1st trimester (Table 2). Participants were more likely to work outside before pregnancy (64.3%) than participants in the 1st trimester (48.2%) or in the 2nd trimester (45.0%). Compared to those in pre-pregnancy, women in the early pregnancy reduced physical activity sharply and in the second trimester basically returned to physical activity level as it before pregnancy. Dietary fat intake, protein intake, animal protein intake and vegetable protein intake in the mid pregnancy were higher than those in the pre-pregnancy or in the early pregnancy, while their energy intake from carbohydrate was lower than those in the pre-pregnancy and in the early pregnancy. Not surprisingly, consumption of meats, eggs, dairy and dairy products, fish and shrimp were greatest in the mid pregnancy as well as beans and nuts consumption.

**Table 2:** Anthropometric, behaviors, and nutritional data^a^ at the pre-pregnancy and during pregnancy in participants (*n* = 6,299).

	**Pre-pregnancy**	**1st trimester**	**2nd trimester**
**ANTHROPOMETRIC DATA**			
Body weight (kg)	53.7 (5.3)	55.1 (4.6)	61.3 (5.7)
Gestational weight gain (kg) during each trimester	–	1.2 (2.1)	5.5 (2.7)
**BEHAVIORS**			
Multivitamin use [*n* (%)]	3,716 (59.0)	6,179 (98.1)	5,026 (79.8)
Work outside [*n* (%)]	4,050 (64.3)	3,036 (48.2)	2,834 (45.0)
Physical activity (MET-h/wk)^b^	18.6 (10.4)	13.2 (8.7)	17.2 (10.2)
Self-reported passive smoking [*n* (%)]	3,149 (50.0)	2,103 (33.4)	1,700 (27.0)
Self-reported active smoking [*n* (%)]	170 (2.7)	31 (0.5)	0 (0.0)
Self-reported alcohol drinking [*n* (%)]	1,650 (26.2)	120 (1.9)	19 (0.3)
**DIETARY INTAKE**^c^			
Total Energy (kcal/d)	1658.2 (516.9)	1639.8 (472.0)	1986.9 (555.3)
Carbohydrate (% of energy)	52.6 (8.7)	52.7 (8.2)	48.6 (7.6)
Fat (% of energy)	31.9 (8.0)	31.7 (7.3)	33.6 (6.5)
Protein (% of energy)	15.5 (2.3)	15.6 (2.3)	17.8 (2.6)
Animal protein (% of energy)	9.2 (2.8)	9.3 (2.8)	11.2 (2.7)
Vegetable protein (% of energy)	6.3 (1.6)	6.3 (1.6)	6.6 (1.6)
Meats (g/d)	94.0 (27.0)	83.9 (21.9)	128.5 (24.2)
Eggs (g/d)	49.8 (3.9)	41.6 (5.7)	64.7 (4.1)
Dairy and dairy products (g/d)	217.9 (27.7)	259.1 (21.5)	377.1 (23.9)
Fish and shrimp (g/d)	23.9 (6.6)	21.0 (8.1)	75.5 (5.9)
Beans (g/d)	15.9 (20.8)	17.5 (33.3)	18.4 (29.4)
Nuts (g/d)	9.7 (20.6)	19.2 (17.0)	23.3 (16.7)

a *Values are means (SD)*.

b Metabolic equivalent hours of activity per week.

c *All nutritional data represent crude values*.

### Relative risk for GDM by the dietary protein intake before pregnancy and during the 1st trimester and the 2nd trimester

Higher intake of total protein, animal protein and vegetable protein before pregnancy and in the early pregnancy were not associated with risks for GDM (Table 3). In mid pregnancy, higher total protein intake or animal protein intake was associated with higher risks for GDM: women with highest total protein intake or animal protein intake had an approximately 92% (67%) higher risk for GDM than those in the lowest total protein intake tertile or animal protein intake tertile. Vegetable protein intake in the 2nd trimester was not related to GDM risk.

**Table 3:** Dietary protein intakes before and during pregnancy and risk of gestational diabetes^a^ (*n* = 6,299).

	**Protein intake in tertile**		
**Variable**	**T1**	**T2**	**T3**	***p***
**PRE-PREGNANCY**				
**Total protein**
Median (% energy/d)	13.40	15.36	17.42	–
Model 1	1.00	1.91 (1.04–3.56)	1.17 (0.61–2.27)	0.08
Model 2	1.00	1.86 (1.01–3.49)	1.14 (0.60–2.21)	0.10
Model 3	1.00	2.07 (1.09–4.01)	1.59 (0.76–3.39)	0.09
**Animal protein**
Median (% energy/d)	6.69	9.15	11.75	–
Model 1	1.00	1.36 (0.75–2.49)	0.90 (0.48–1.70)	0.37
Model 2	1.00	1.39 (0.76–2.56)	0.90 (0.76–2.56)	0.33
Model 3	1.00	1.60 (0.83–3.13)	1.28 (0.56–2.92)	0.36
**Vegetable protein**
Median (% energy/d)	4.89	6.21	7.79	–
Model 1	1.00	1.09 (0.59–2.05)	1.33 (0.73–2.46)	0.64
Model 2	1.00	1.11 (0.60–2.09)	1.33 (0.73–2.47)	0.64
Model 3	1.00	1.21 (0.62–2.37)	1.45 (0.69–3.07)	0.63
**EARLY PREGNANCY**				
**Total protein**
Median (% energy/d)	13.69	15.44	17.47	–
Model 1	1.00	1.04 (0.56–1.95)	1.38 (0.75–2.53)	0.52
Model 2	1.00	0.97 (0.51–1.83)	1.31 (0.72–2.43)	0.55
Model 3	1.00	0.79 (0.41–1.54)	0.89 (0.43–1.86)	0.79
**Animal protein**
Median (% energy/d)	6.95	9.22	11.52	–
Model 1	1.00	0.89 (0.50–1.67)	1.19 (0.66–2.17)	0.65
Model 2	1.00	0.86 (0.46–1.61)	1.13 (0.62–2.07)	0.68
Model 3	1.00	0.62 (0.30–1.27)	1.07 (0.75–1.69)	0.54
**Vegetable protein**
Median (% energy/d)	4.75	6.10	7.71	–
Model 1	1.00	0.74 (0.41–1.36)	0.71 (0.39–1.30)	0.48
Model 2	1.00	0.80 (0.44–1.47)	0.73 (0.40–1.34)	0.58
Model 3	1.00	0.94 (0.48–1.81)	0.90 (0.43–1.86)	0.96
**MID PREGNANCY**				
**Total protein**
Median (% energy/d)	15.51	17.45	20.24	–
Model 1	1.00	1.24 (1.03–2.40)	2.11 (1.15–2.96)	0.04
Model 2	1.00	1.19 (1.01–2.31)	2.07 (1.12–2.90)	0.04
Model 3	1.00	1.18 (1.02–2.37)	1.92 (1.10–3.14)	0.04
**Animal protein**
Median (% energy/d)	8.36	11.10	13.76	–
Model 1	1.00	1.45 (1.13–2.35)	1.83 (1.17–2.49)	0.05
Model 2	1.00	1.48 (1.14–2.44)	1.81 (1.16–2.47)	0.04
Model 3	1.00	1.38 (1.12–2.93)	1.67 (1.19–2.93)	0.03
**Vegetable protein**
Median (% energy/d)	5.16	6.45	8.00	
Model 1	1.00	0.77 (0.41–1.43)	1.04 (0.57–1.88)	0.60
Model 2	1.00	0.76 (0.41–1.42)	1.01 (0.56–1.42)	0.61
Model 3	1.00	0.87 (0.46–1.67)	1.33 (0.69–2.58)	0.42

a *Values are medians and intakes were calculated as the percentage of energy by tertile. Model 1: crude model. Model 2: additionally adjusted for age, pregravid BMI, and parity. Model 3: additionally adjusted for family history of diabetes (yes or no), current smoking (yes or no), PCOS (yes or no), family income, total energy intake, fat intake, carbohydrate intake, and physical activity*.

### Relative risk for GDM by the major dietary protein sources in the mid pregnancy

In our population, animal protein accounted for the majority and the major source of animal protein is from meats, fish and shrimp, eggs, dairy, and dairy products. The main contributors to vegetable protein were beans and nuts.

**Figure 3:**
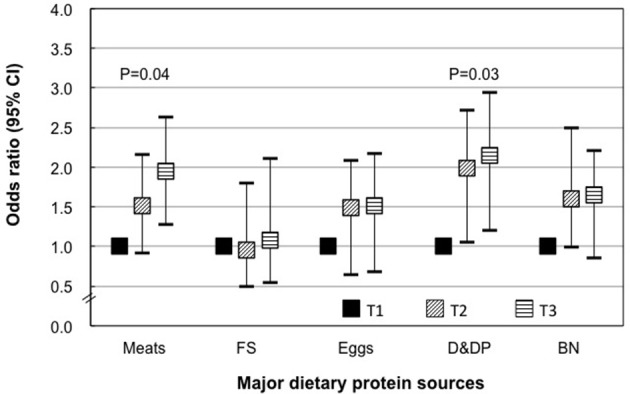
Specific dietary protein intakes during mid pregnancy and risk of gestational diabetes. T, tertile; FS, fish and shrimp; D&DP, dairy and dairy products; BN, beans and nuts. Data shown are OR and 95% confidence interval. Models were adjusted for additionally adjusted for age, pregravid BMI, parity, family history of diabetes (yes or no), current smoking (yes or no), PCOS (yes or no), family income, total energy intake, fat intake, carbohydrate intake, and physical activity.

In the mid pregnancy, higher meat consumption, dairy, and dairy products were associated with higher risk for GDM: participants with highest meat consumption or dairy consumption had an approximately 95% (115%) higher odds for GDM than those in the lowest tertile of meat or dairy consumption (Figure 3).

## Discussion

Our study indicated that higher dietary protein intake and animal protein intake in mid-pregnancy, but not in early pregnancy or before pregnancy, were associated with risks for GDM in a Chinese population. In addition, the main dietary protein sources in 2nd trimester such as meats or dairy products were significantly associated with a higher risk of GDM.

To our knowledge, this is the first report on dietary changes before and during pregnancy among Chinese women. Compared to the dietary protein intake before pregnancy, a clear increase was seen in pregnancy, especially it in the 2nd trimester, among our sample. This is in line with the data from Sweden women (*n* = 50) (35) (14.4% of energy vs. 16.8% of energy) and Portugal mother (*n* = 249) (34) (17.6% of energy vs. 18.4% of energy). In addition, our finding of the main dietary source of protein are broadly consistent with that observed among British in The Southampton Women's Survey (*n* = 1,490) and Hungarian women (*n* = 349) (33, 36). The reason for this result may be due to physiological changes, traditional beliefs and dietary recommendations during pregnancy (32).

Physical activity is a well-established risk factor for the development of GDM (42). In present study, we were not surprised to find that with the increase of gestational age, the work of pregnant women was significantly reduced, and physical activity during pregnancy (especially in the first trimester) was also reduced. The effect of mid-pregnancy protein on the incidence of GDM may be enhanced by decline physical activity. However, after analysis, there was still a significant risk of GDM associated with high levels of protein even when the decline in physical activity was accounted for.

Our data do in addition suggest that the relation of dietary protein intake to risk of GDM is particularly pronounced in the time window closer to GDM diagnosis, at least for Chinese women, i.e., only protein intake during mid-pregnancy rather than in pre-pregnancy or in early pregnancy is relevant for risk of GDM. Pregnancy is characterized by a series of metabolic changes, including the development of physiological insulin resistance. During early pregnancy, glucose tolerance is normal or even slightly improved, and peripheral sensitivity to insulin and hepatic basal glucose production is normal (43), however, in later gestation, maternal adipose tissue depots decline, while post-prandial free fatty acid levels increase, and insulin mediated glucose disposal worsens by 50–70% compared with pre-pregnancy (12). Dietary proteins which may modulate insulin sensitivity (15) are thus of specific relevance on the glucose homeostasis for the pregnant women, with respect to the physiological insulin resistance. However, data from the NHS II Cohort Study (19) suggested that dietary protein intake or animal protein intake before pregnancy was related to higher GDM risk. The reason of the inconsistence of our findings with NHS II data might be the intake level among study population (15.5% in our participants vs. 19.14% in US women). The other study in Guangzhou China inconsistently demonstrated that protein-rich pattern was not associated with the risk of GDM, which may be attributed to the different dietary patterns in South China and Southwest China. In addition, unlike our study of single nutrients, there are interactions between nutrients in dietary patterns (44).

In the current study, we discovered that an increase in the intake of animal protein during the 2nd trimester was associated with higher risk of GDM which is in line with previous findings on a relevance of protein intake in mid pregnancy for GDM confirming this for both 1,247 women from Singapore (17) and 1,261 pregnant women from China (18). Although the mechanisms between high dietary animal protein intake and GDM risks are unclear, the observed association with GDM is biologically plausible. It could be caused by several nutrients, such as iron and amino acids. An overload of iron can cause insulin resistance leading to increase in the production of hepatic glucose (45, 46), subsequently reducing the secretion of insulin (47). Considering that an increase in the consumption of animal protein may contribute to increased body iron load, the association between a higher animal protein intake and gestational diabetes in the mid-pregnancy may be partly explained markers of body iron load. Second, the ingestion of meals rich in animal protein can cause a significant increase in the plasma concentration of BCAAs (branched-chain amino acids) with a notably altered metabolism as observed in conditions characterized by insulin resistance, insulin deficiency, or both (48).

Moreover, we found that higher consumption of meat and dairy products, two major dietary sources of animal protein, were associated with higher risk of GDM. Meat is a principal food source of dietary protein and its potential mechanisms may involve advanced glycation end-products (49) or gamma-glutamyl-transferase (50) with high meat intake. Dairy products have been identified as potent insulin secretagogues, as their consumption stimulates acute hyperinsulinemia (51). For patients with hyperglycemia and type 2 diabetes, hyperinsulinemia caused by consumption of dairy products may be beneficial or even protective for regulating blood glucose levels (52). However, long-term hyperinsulinemia from the consumption of dairy products may have adverse long-term effects on healthy individuals, including insulin resistance (53).

Given that the substantial relevance of GDM for medium and long-term health of both mother and offspring, the influences of dietary protein intake and animal protein intake as well as meat and dairy consumption on GDM as those observed in our study will identify the modifiable dietary risk factors and critical time window which contribute to GDM development and thus have important public health implications in view of the high GDM prevalence in the Chinese population.

Some limitations of our study should be mentioned. Southwestern China is home for many ethnic minorities. We could not examine the association in each ethnic minority group. However, involvement of such an ethnically diverse population may increase generalizability of our findings. Secondly, there is still controversy in the diagnostic criteria of GDM. In the current study, GDM was assessed according to the IADPSG criteria which has been endorsed by the American Diabetes Association (1) and Chinese Ministry of Health (8). However, how the diagnostic criteria will affect the observed association between dietary protein intake and GDM warrants further investigation. Thirdly, assessing diet based on the memory-based questionnaire has its limitations, but we think the best which could be done in the circumstances. Instead keeping a food diary and assessing the protein and another nutrients intake would be a better way, but very cumbersome and difficult. Finally, we cannot rule out the possibility of residual confounding from unmeasured or unknown factors.

The strengths of this analysis include its prospective nature data, large sample size, high response rate, and detailed measurement of both dietary data and clinical measures in conjunction with the ability to adjust for a number of major potential confounders. Notably, unlike previous studies on the association between protein intake and GDM in only one specific period (pre-pregnancy or mid pregnancy), we carefully chose our study period to cover the time period ranging from the year preceding pregnancy to birth, which make it possible to investigate the critical time window of the impact of dietary protein intake on GDM risk. Specifically, we used the same sample for analysis of the different potentially critical periods, therefore minimizing the heterogeneity among different women. Finally, compared to the 24-h recalls, the validated FFQs might better reflect the habitual dietary intake over a period of time.

In conclusion, our study indicated that higher dietary intakes of total protein and animal protein in mid-pregnancy were associated with an increased risk of GDM among pregnant Chinese women. The findings in this study can be defined and examined by random prospective studies in the future.

## Author contributions

YL performed the analyses and wrote the manuscript. YG, XZ, DY, DZ, LQ, and RZ coordinated the study centers. WB and GC supervised the analyses. GC conceived the project. All authors critically reviewed the manuscript for important intellectual content.

### Conflict of interest statement

The authors declare that the research was conducted in the absence of any commercial or financial relationships that could be construed as a potential conflict of interest.
